# Triflumuron Effects on the Physiology and Reproduction of* Rhodnius prolixus* Adult Females

**DOI:** 10.1155/2016/8603140

**Published:** 2016-10-16

**Authors:** Bianca Santos Henriques, Fernando Ariel Genta, Cícero Brasileiro Mello, Lucas Rangel Silva, Thaís Franco Codogno, Alyne F. R. Oliveira, Lourena Pinheiro Marinho, Denise Valle, José Bento Pereira Lima, Denise Feder, Marcelo Salabert Gonzalez, Patricia Azambuja

**Affiliations:** ^1^Laboratório de Bioquímica e Fisiologia de Insetos, Instituto Oswaldo Cruz (FIOCRUZ), Pavilhão Leônidas Deane, Sala 207, Avenida Brasil 4365, Manguinhos, 21040-900 Rio de Janeiro, RJ, Brazil; ^2^Instituto Nacional de Ciência e Tecnologia em Entomologia Molecular, Avenida Carlos Chagas Filho 373, Bloco D, Centro de Ciências da Saúde Bloco D-SS, Sala 05, Cidade Universitária, 21941-902 Rio de Janeiro, RJ, Brazil; ^3^Laboratório de Biologia de Insetos, Departamento de Biologia Geral, Instituto de Biologia, Universidade Federal Fluminense (UFF), Morro do Valonguinho, S/N^0^, 24001-970 Niterói, RJ, Brazil; ^4^Laboratório de Biologia Molecular de Flavivírus, Instituto Oswaldo Cruz (FIOCRUZ), Pavilhão Leônidas Deane, Sala 317, Avenida Brasil 4365, Manguinhos, 21040-900 Rio de Janeiro, RJ, Brazil; ^5^Laboratório de Fisiologia e Controle de Artrópodes Vetores, Instituto Oswaldo Cruz (FIOCRUZ), Avenida Brasil 4365, Manguinhos, 21045-900 Rio de Janeiro, RJ, Brazil; ^6^Laboratório de Entomologia, Instituto de Biologia do Exército, Rua Francisco Manuel 102, Triagem, 20911-270 Rio de Janeiro, RJ, Brazil

## Abstract

We evaluated the efficacy of the growth regulator triflumuron (TFM) in inducing mortality and disrupting both oviposition and egg hatching in* Rhodnius prolixus* adult females. TFM was administered via feeding, topically or by continuous contact with impregnated surfaces. Feeding resulted in mild biological effects compared with topical and impregnated surfaces. One day after treatment, the highest mortality levels were observed with topical surface and 30 days later both topical and impregnated surfaces induced higher mortalities than feeding. Oral treatment inhibited oviposition even at lower doses, and hatching of eggs deposited by treated females was similarly affected by the three delivery modes. Topical treatment of eggs deposited by nontreated females significantly reduced hatching. However, treatment per contact of eggs oviposited by untreated females did not disrupt eclosion. Additionally, oral treatment increased the number of immature oocytes per female, and topical treatment reduced the mean size of oocytes. TFM also affected carcass chitin content, diuresis, and innate immunity of treated insects. These results suggest that TFM acts as a potent growth inhibitor of* R. prolixus* adult females and has the potential to be used in integrated vector control programs against hematophagous triatomine species.

## 1. Introduction

The insecticide activity of the benzoylphenylurea (BPU) family was discovered in the 1970s by accident during herbicidal screening, after which Diflubenzuron, its first synthesized analogue, started to be commercialized [1]. BPU substances are composed of one benzoyl ring, one aniline ring, and urea bridge, each one playing different roles in activity, varying only in its substituents [2].

There are 15 BPU compounds being commercialized, with over 10000 described [2], which have been used as Insect Growth Regulators (IGRs) against a wide range of crop, pasture, and forest pests [3, 4]. They represent a promising control method for insect populations, able to avoid the harmful side effects of conventional insecticides on both the environment [5, 6] and human health [7].

BPU substances appear to act by inhibiting the chitin synthesis pathway during insect development [8]. This reduction results in mortality, moulting failure, and/or malformations of the cuticle [9–15]. In addition, adults emerging from BPU-treated larvae display physiological changes, which ultimately lead to decreased physical and reproductive fitness [3]. BPU substances are relatively selective toward arthropods and safe to humans since chitin is absent in vertebrates [16]. Because of that, they are often preferred to broad-spectrum insecticides when control operations are conducted in the field [17, 18].

Chagas disease [19, 20] affects 18 million people in South America [21]. It is transmitted by hematophagous insects of the subfamily Triatominae (order Hemiptera, family Reduviidae) such as* Rhodnius prolixus*. Additionally,* R. prolixus* is a traditional model for physiological and parasite-vector interaction studies [22].

Although several reports describe the effects of BPU substances against disease vectors [23–30], little is known about their implication in the biology and reproductive fitness of Chagas disease vectors [31]. We previously confirmed the effectiveness of triflumuron (TFM) against* R. prolixus* nymphs [32]. The aim of this work is to extend these studies and determine the biological and physiological effects of TFM (Starycide® sc 480 Bayer) on* R. prolixus* adult females. We investigated the effects of different TFM doses administered by three different delivery modes: feeding and topical and continuous contact with impregnated surfaces. Additionally, we investigated some TFM-induced physiological features which might be leading to inhibition of reproduction and insect death. These features include oogenesis, chitin content, diuresis, and prophenoloxidase activation. Altogether, these results might be valuable for evaluation of perspectives for integrated vector control programs against blood sucking triatomine species.

## 2. Material and Methods

### 2.1. Insects

Adult females of* Rhodnius prolixus* were reared in laboratory conditions at 28°C and relative humidity of 60–70% [32]. Randomly chosen insects were then allowed to feed upon a membrane apparatus [33] and submitted to biological assays (see Section 2.3).

### 2.2. Chemicals

We used Starycide sc 480, containing 48 g of TFM per 100 mL, in the experiments. L-3,4-Dihydroxyphenylalanine (L-DOPA) was purchased from Sigma (Cat. no. D9628). Unless mentioned, all solutions were prepared with deionized water (Milli-Q®, Millipore). Other reagents used were of analytical grade.

### 2.3. Biological Assays

Following ecdysis, we fed fifth-instar female nymphs of* R. prolixus* with defibrinated rabbit blood and separated them until they reached adult stage. After metamorphosis, we submitted primiparous females to three different TFM treatments (see the following). In the control group they were fed with defibrinated rabbit blood only. Oral treatment consisted of adding TFM to the blood meal at concentrations from 0.1 to 10 *μ*L per mL.

Topical treatment consisted of direct application of Starycide to the dorsal surface of each insect abdomen in doses ranging from 0.1 to 10 *μ*L immediately after feeding.

For the impregnated surface treatment, we applied Starycide evenly onto a filter paper placed at the bottom of a Petri dish (⌀ 9 cm) in order to obtain final concentrations ranging from 0.1 to 10 *μ*L/cm^2^ after evaporation of solvent.

Immediately after feeding and/or treatment, we placed the adult insects (control and treated groups) in Petri dishes (⌀ 9 cm) by a proportion of one untreated male to 10 treated females and followed them during 30 days. We used only fully engorged insects after only one blood meal throughout the experiments. We replaced the untreated males in case of death. There was no additional surface inside the Petri dish, so the insects were forced to be in contact with the treated paper.

We recorded the biological evaluation of the different treatments by weight of ingested blood, amount of excretion, toxicity (i.e., short and long term mortality, represented by 24 h and 30 days after treatment), malformations of the cuticle, oviposition, and egg hatching.

In another experiment, nonmated adult females were fed with blood containing 1 *μ*L TFM/mL or treated topically with 1 *μ*L TFM after feeding as described above. These insects were weighed daily during 15 days. Controls received no TFM.

Additionally, one-day-old eggs obtained from untreated healthy primiparous females were submitted to topical treatment using 0.1 to 1 *μ*L of TFM applied directly per egg. Eggs deriving from untreated mothers were also submitted to continuous contact with TFM impregnated surfaces (0.1 to 10 *μ*L/cm^2^) as described above. The eggs were then observed during 30 days to evaluate hatching. All experiments were repeated at least in triplicate with batches of 10 insects or 10 eggs per group depending on the experiment done.

### 2.4. Chitin Measurements

We measured chitin according to [34]. After oral (3 *μ*L/mL blood) or topical (3 *μ*L/insect) treatments with TFM, we kept females for 5 days in Petri dishes with an untreated male (mated group) as described above. Another experiment was set without the inclusion of a male in the Petri dish (nonmated group). Insects were ice-anesthetized and then dissected in cold 0.9% NaCl. Gut, ovaries, and fat body were removed and the remaining tissues (named carcass) were then homogenized in liquid nitrogen with the aid of a ceramic mortar and pestle. Powdered tissues from three female insects were then resuspended in 2 mL of water and then 200 *μ*L was centrifuged at 21,000 ×g for 5 minutes at room temperature. We discarded the supernatants, resuspended the pellets in 3% (w/v) sodium dodecyl sulphate, and then incubated them at 100°C for 15 minutes. Samples were cooled and centrifuged as above and supernatants were discarded. Pellets were washed as above with 200 *μ*L water and then resuspended with 150 *μ*L of 2.1 M KOH. Samples were then heated at 130°C for 1 h. After cooling, 400 *μ*L of 75% (v/v) ethanol was added and samples were kept in ice for 15 minutes. Then 60 *μ*L of a Celite 545 suspension (supernatant of 1 g suspension in 12.5 mL 75% ethanol, resting for 2 minutes) was added. Samples were centrifuged and the pellets were washed with 600 *μ*L of 40% ethanol and then 600 *μ*L of water as above. Those pellets (insoluble chitosan) were frozen at −20°C or kept at 4°C until assay.

Pellets were resuspended in 125 *μ*L water, then 125 *μ*L of NaNO_2_ 5% (w/v) plus 125 *μ*L of KHSO_4_ 5% (w/v) was added, and samples were incubated at room temperature for 15 minutes. A control tube consisting of 125 *μ*L water instead of sample pellet suspension was included in this step. After centrifugation (1,500 ×g, 2 minutes, 4°C) two 150 *μ*L aliquots were withdrawn as replicates and each one was combined to 50 *μ*L of ammonium sulfamate 12.5% (w/v) and 50 *μ*L 12.5% (w/v) MBTH (3-methyl-benzo-2-thiazolone hydrazone). MBTH was prepared daily. Samples were then incubated at 100°C for 3 minutes. After cooling, 50 *μ*L of FeCl_3_·6H_2_O 0.83% (w/v) was added and then samples were incubated for 25 minutes at room temperature. Aliquots with 200 *μ*L were transferred to 96-well microplates and absorbance at 650 nm was measured. A standard curve was performed using a 3.3 *μ*g/*μ*L colloidal chitin suspension prepared according to [35] and used to calculate the amount of chitin recovered from each insect.

### 2.5. Phenoloxidase Assays

Phenoloxidase assays were performed as previously described [36]. Insects were submitted to oral treatment (0–0.5 *μ*L TFM/mL blood) and, 7, 12, or 16 days after feeding, hemolymph was withdrawn using 10 *μ*L glass tubes. Hemolymph samples were diluted 10 times with 10 mM sodium cacodylate buffer pH 7.4 containing 10 mM CaCl_2_ and then 25 *μ*L was combined with 10 *μ*L of the same buffer above (spontaneous activity) or 10 *μ*L of a 1 mg/mL trypsin solution in the same buffer (total activity). Reaction mixtures were then incubated at 37°C for 20 minutes and phenoloxidase measurements were then started by the addition of 25 *μ*L of a saturated solution of L-DOPA (4 mg/mL). Dopachrome formation was followed by continuous absorbance measurements at 490 nm at 37°C for 60 minutes (Spectra Max, Molecular Devices).

### 2.6. Oocyte Measurement and Observation of Internal Anatomy

We treated adult females of* R. prolixus* with Starycide orally (3 *μ*L TFM/mL blood) or topically (3 *μ*L/insect) as described above. Control insects received no treatment. Five days after treatment, insects were dissected and oocytes were counted and photographed with a millimeter paper for scale. For measurement of oocyte length, photos were analyzed using the programs Photoshop CS6 Extended or ImageJ. For internal anatomy comparison, photos were taken using a stereomicroscope Leica MZ6 with 10x magnification.

### 2.7. Data Analysis

The significance of results was analyzed using ANOVA and Tukey's test [37]. Differences between treated and control insects or eggs were considered nonstatistically significant when *p* > 0.05. Probability levels are specified in the text.

## 3. Results

### 3.1. Effects of Feeding Treatment with TFM on* R. prolixus* Females

The biological data of* R. prolixus* adult females after oral treatment with TFM are presented in Table 1. It is important to notice that control insects showed a mortality of 9% one day after feeding, with a mortality of 19% after 30 days. Nevertheless, TFM treatment at all doses (0.1–10 *μ*L/mL blood) induced significantly higher mortality levels than controls, either one day after feeding, with mortalities ranging from 23 to 46% (*p* < 0.05, unpaired *t*-test), or after 30 days (50–76%, *p* < 0.01, unpaired *t*-test).

Control females laid an average of 56 ± 1 eggs, with a hatching rate of 70% (Table 1). All TFM doses tested (0.1–10 *μ*L/mL blood) resulted in significantly lower numbers of eggs, ranging from 20 to 40 eggs per female (*p* < 0.05, unpaired *t*-test).

Egg hatching was also affected by oral treatment with TFM (Table 1). Treatment with 0.1 *μ*L/mL blood did not affect this parameter, but doses equal or above 0.5 *μ*L/mL resulted in significantly lower hatching ratios, equal to or below 16% (*p* < 0.001, unpaired *t*-test).

### 3.2. Effects of Topical Treatment with TFM on* R. prolixus* Females and Eggs

The biological data of* R. prolixus* adult females and eggs after topical treatment with TFM are presented in Table 2. As observed in the previous experiments, controls presented some mortality 1 day after feeding (6 ± 3%), which increased to 23 ± 3% after 30 days. All doses of TFM (0.1–10 *μ*L/insect) resulted in significant higher mortality levels than controls, either 1 day after treatment, ranging from 50 to 90% (*p* < 0.01, unpaired *t*-test), or after 30 days, above 86% (*p* < 0.001, unpaired *t*-test).

Control insects laid an average of 60 ± 6 eggs/female, with a hatching ratio of 70% (Table 2). A strong effect was also observed in the number of eggs laid by females, which decreased to 40 eggs/female with 0.5 *μ*L TFM/insect (*p* < 0.05, unpaired *t*-test) and eventually to 20 eggs/female with 10 *μ*L TFM/insect (*p* < 0.001, unpaired *t*-test). A similar effect was also observed in egg hatching, with ratios decreasing to 26% (0.5 *μ*L TFM/insect; *p* < 0.001, unpaired *t*-test) or even 9% (10 *μ*L TFM/insect; *p* < 0.001, unpaired *t*-test).

The effect of topical treatment with TFM administered directly to eggs freshly laid was also studied (Table 3). Control eggs have shown hatching rate of 93% and, in this case, a significant reduction to 60% was observed with the lowest dose tested (0.1 *μ*L TFM/egg; *p* < 0.05, unpaired *t*-test). Higher doses resulted in lower hatching ratios, reaching the observed minimum of 10% with 1 *μ*L TFM/egg (*p* < 0.01, unpaired *t*-test).

### 3.3. Effects of Impregnated Surface Treatment with TFM on* R. prolixus *Females and Eggs

The biological data of* R. prolixus* adult females and eggs after continuous treatment with TFM are presented in Table 4. In these experiments, mortalities in the control group were 6 ± 3% and 16 ± 1% after 1 and 30 days of treatment, respectively. Even the lowest TFM dose tested (0.1 *μ*L/cm^2^) resulted in significant effects on mortality, with respective rates of 36% (*p* < 0.05, unpaired *t*-test) and 86% (*p* < 0.001, unpaired *t*-test) after 1 and 30 days of treatment. Higher doses resulted in somewhat higher mortalities, with maximum observed values of, respectively, 63% (*p* < 0.01, unpaired *t*-test) and 96% (*p* < 0.001, unpaired *t*-test) at the dose of 10 *μ*L/cm^2^, after 1 and 30 days of treatment.

Control insects, in these experiments, laid an average of 59 ± 2 eggs/female, with a hatching ratio of 70%. Oviposition was significantly affected by TFM continuous treatment, even at the lowest dose tested (0.1 *μ*L/cm^2^), with a decrease in number of eggs to 43 ± 5 eggs/female (*p* < 0.05, unpaired *t*-test). Higher doses resulted in stronger inhibitions, reaching the observed minimum of 17 ± 1 eggs/female at 10 *μ*L/cm^2^ (*p* < 0.001, unpaired *t*-test). The hatching of eggs laid by treated females was not significantly affected by 0.1 *μ*L/cm^2^ (*p* > 0.05, unpaired *t*-test), but was decreased by doses equal to or higher than 0.5 *μ*L/cm^2^, reaching the observed minimum of 3.6 ± 0.3% (*p* < 0.001, unpaired *t*-test).

The effect of the continuous contact of eggs laid by untreated females with TFM impregnated surfaces was also tested. Interestingly, no significant effect on egg hatching was observed after continuous TFM treatment (Table 5). However, only few of the 1st-instar larvae that emerged were able to survive after eggs eclosion (data not shown).

### 3.4. Effects of TFM Treatment of* R. prolixus* Adult Females on Oocyte Development

The effects of oral and topical TFM treatment in* R. prolixus* oocyte development are shown in Table 6. Control insects, 5 days after blood feeding, showed an average of 5 ± 1 oocytes/female, with an average oocyte size of approximately 2 mm. Oral treatment with TFM had no significant effects on average oocyte size (*p* > 0.05, unpaired *t*-test) but resulted in a significant higher number of oocytes per female (9 ± 2, *p* < 0.05, unpaired *t*-test). Nevertheless, topical treatment had no significant effect on the number of eggs per female (*p* > 0.05, unpaired *t*-test) and resulted in significantly smaller oocytes (1.78 ± 0.06 mm, *p* < 0.05, unpaired *t*-test).

### 3.5. Effect of TFM Treatment on Chitin Content of* R. prolixus* Females

The effect of oral and topical treatment with TFM on the chitin content of* R. prolixus* adult females is described in Table 7. We tested the effects of TFM on chitin contents of mated and nonmated females. The amount of chitin was quantitated by the colorimetric technique of [34], which was not sensitive enough for reliable detection of chitin in the internal organs of* R. prolixus*. Therefore, we limited our comparisons to the chitin amounts that could be recovered from insect carcasses, which consisted of the remaining tissues of dissected insects after the removal of gut, ovaries, and fat body.

Mating resulted in a significant increase of carcass chitin contents in control females (*p* < 0.01, unpaired *t*-test). The amount of carcass chitin in nonmated females was significantly reduced by both TFM treatments (*p* < 0.05, unpaired *t*-test), with a stronger effect in the topical treatment. However, TFM topical treatment did not significantly change the amount of carcass chitin in mated females (*p* > 0.05, unpaired *t*-test), which was significantly reduced by the oral treatment only (*p* < 0.01, unpaired *t*-test).

### 3.6. Effect of TFM Treatment on Diuresis of* R. prolixus* Adult Females

To have a better understanding of mechanisms leading to mortality in TFM-treated adult female* R. prolixus*, we followed diuresis after oral and topical treatment. In* R. prolixus*, weight increases several times during a blood meal and slowly decreases during digestion. In the first days after a blood meal, this decrease in weight is mainly caused by the quick excretion of water during haemolysis and concentration of gut contents. For this reason, weight measurements after a blood meal can be used as an indication of effective diuresis.

**Figure 1:**
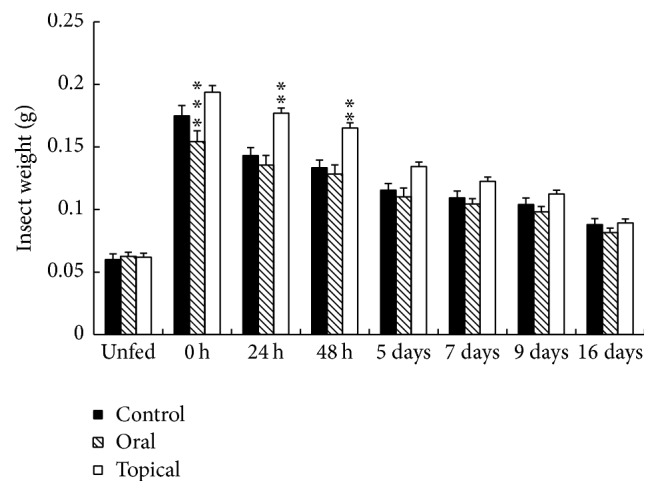
Effect of oral or topical TFM treatment on insect weight of* Rhodnius prolixus* adult females after blood feeding. Control insects were fully engorged on defibrinated rabbit blood only, whereas experimental insects were fed with blood containing 1 *μ*L TFM/mL blood (oral) or directly received 1 *μ*L TFM on the dorsal surface of the abdomen immediately after feeding (topical). Insects were weighted before (unfed) and at different times after blood feeding. Values are means ± SEM of one experiment with 18 insects each. Asterisk marks denote differences between control and experimental groups (unpaired *t*-test, ^*∗∗*^ *p* < 0.01, ^*∗∗∗*^ *p* < 0.001).

Treatment with TFM using oral or topical delivery resulted in different effects on blood ingestion (Figure 1, 0 h). Oral treatment resulted in significant less weight gain than that observed in control insects (*p* < 0.001, unpaired *t*-test). After 24 h, the weight of control insects was indistinguishable from the mean weight of orally treated ones. Nevertheless, insects treated topically with TFM remained significantly heavier than controls up to 48 hours after feeding (*p* < 0.01, unpaired *t*-test). After 5 days, all groups presented similar average weights (*p* > 0.05, unpaired *t*-test). These results suggest that insects treated topically with TFM have impaired diuresis in the first two days after blood ingestion. In contrast, protein digestion after 2 days is negligible, and the whole digestive process in* R. prolixus* final stages takes 2-3 weeks [36]. In this respect, TFM effects on blood digestion are less likely because weight loss after 5 days is similar among the three experimental groups.

**Figure 2:**
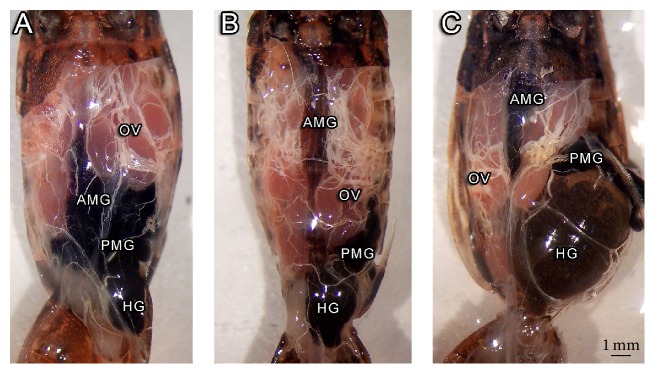
Representative images of internal tissues of* Rhodnius prolixus* adult females after different treatments with TFM. (A) Control insects. (B) Insects fed with defibrinated rabbit blood containing TFM at 1 *μ*L/mL (oral treatment). (C) Insects received 1 *μ*L TFM on the dorsal surface of the abdomen immediately after feeding (topical). OV: ovaries; AMG: anterior midgut; PMG: posterior midgut; HG: hindgut.

The suggestion from data of Figure 1 that TFM topical treatment resulted in an impaired diuresis led us to analyze the internal anatomy of treated insects (Figure 2). Control insects and insects that received TFM orally slightly differ in general internal anatomy, with anterior midgut, posterior midgut, and hindgut showing similar placement and size (Figures 2(A) and 2(B)). However, insects topically treated with TFM showed a clear enlargement of the hindgut, with displacement of the posterior midgut (Figure 2(C)).

### 3.7. Effect of Oral TFM Treatment on Phenoloxidase Activity of* R. prolixus* Females

Another physiological aspect we followed to better understand the effects of TFM treatment leading to adult female mortality was the prophenoloxidase (PPO) activation system.

This was performed by measuring phenoloxidase (PO) activity in the hemolymph. PO is secreted as a zymogen, prophenoloxidase (PPO), which is activated during infection by pathogens. PPO levels, which we measured as total PO activity (see Section 2 for details), are normally constitutive, being regulated by hormonal and developmental processes. However, PO activity, which we measured as spontaneous activity (see Section 2 for details), is sharply increased after an injury or infection. We hypothesized that insects treated with TFM, with a lower chitin content in the carcass, could be more exposed to infections and present a higher PO activity in the hemolymph.

**Figure 3:**
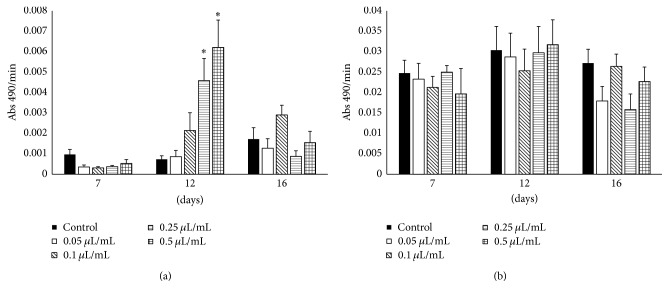
(a) Spontaneous and (b) total phenoloxidase (PO) activities in the hemolymph of* Rhodnius prolixus* adult females orally treated with different doses (0.05–0.5 *μ*L/mL blood) of TFM. Controls were engorged with defibrinated rabbit blood only. Experimental insects were engorged with blood containing TFM and PO activities were measured in the hemolymph 7, 12, or 16 days after feeding. Figures correspond to enzymatic activities measured in 5 samples obtained from one insect each. Asterisk marks denote differences between control and experimental groups (unpaired *t*-test, ^*∗*^ *p* < 0.05).

To test this hypothesis, we orally treated* R. prolixus* adult females with TFM and measured PO and PPO levels 7, 12, or 16 days after treatment (Figure 3). Seven days after TFM treatment, no significant effects on PO levels were observed. However, 12 days after TFM treatment a significant increase in PO activity was observed with higher doses (>0.25 *μ*L/mL blood, *p* < 0.05, unpaired *t*-test). Sixteen days after feeding with TFM, its effect on PO activity was not detected anymore (Figure 3(a)). This could be related to the high mortality observed with this treatment, since we measured activity only in the survivors that showed no PO response. Another possibility is that insects responded to the treatment and PO activities returned to initial levels. Additionally, no changes in total PO activity were observed independent of time and TFM treatment (Figure 3(b)).

## 4. Discussion

Triflumuron is BPU and inhibits chitin synthesis in developing insects [4, 28]. TFM is nowadays used against both stored products pests and disease vectors [38–48], strongly affecting insect reproduction by disruption of oviposition and/or eclosion of eggs laid by treated females. Its ovicidal effect is achieved either by direct application onto eggs [39, 49, 50] or by treatment of insects at any life stage [25, 51–53]. BPU-treated immature stages and adults derived from them frequently lay fewer and nonviable eggs when compared to their untreated counterparts [54–59]. This occurs by full or partial disruption of both embryogenesis and gonads development of both sexes [51–53]. Moreover, BPU effects vary according to insect species, developmental stage at time of application, kind of compound, mode, and dose of administration [28, 31, 60, 61].

Our data obtained with adult females of* R. prolixus* corroborate previous observations, showing that TFM is able to induce high levels of mortality after treatment [32]. Regardless of the putative differences in the chitin metabolism between male nymphs [32] and adult females (present work), we registered similar mortalities among these groups. This was at 1 and 30 days after treatment for the three delivery modes tested, with the only exception of long term mortality after oral treatment. Male fifth-instar nymphs showed higher mortalities (86–96%) than adult females (50–76%) 30 days after feeding with similar doses of TFM. This is possibly caused by ingestion of larger blood meals by fifth-instar nymphs when compared to adults [62].

Additionally, TFM significantly inhibits both oviposition and hatching of eggs laid by surviving females. Significant mortality levels after treatment were already observed with the lowest dose for the three delivery modes both 1 and 30 days after treatment. However, there were some differences among the three delivery modes in observed mortality levels. One day after TFM administration, oral delivery showed lower mortalities (23–40%) compared with surface contact (36–63%) or topical (50–90%) treatments. This could be a result of TFM excretion or detoxification. It has been shown that gut microbes have an important role in the detoxification of ingested xenobiotics [63], and it has been described that, after a blood meal, there is a huge increase in the bacterial population in* R. prolixus* gut contents [64, 65]. However, our data suggest that oral treatment does not affect diuresis (Figures 1 and 2), so the elimination of TFM in urine after oral treatment should also be considered. This might be an interesting topic for future investigations. Also, the surface contact treatment showed a lower mortality (36–63%) compared with topical one (50–90%), 1 day after treatment. This could be a result of better absorption of TFM through the cuticle in the topical treatment. It is important to consider that the highest dose of TFM used corresponds to 10% of the mass of unfed animals, and this might generate unspecific toxic effects.

When we consider mortality levels 30 days after TFM administration, the oral treatment showed a lower effect (50–76%) than the topical (86–96%) and impregnated surfaces (86–96%) delivery modes. This is possibly due to TFM detoxification at the first days after treatment (see the above). Interestingly, topical and impregnated surfaces treatments showed similar cumulative mortalities after 30 days. This indicates that, in spite of the stronger effect in the first day for the topical treatment, possibly due to better penetration, TFM impregnated surfaces are able to affect the insects during a more prolonged period, suggesting that, under our experimental conditions, environmental degradation of TFM is negligible [66].

Comparing the effects of different delivery modes on oviposition at the lowest doses used, the oral treatment was more effective (30 eggs/female) than topical (56) or impregnated surface (43). At higher doses, all treatments showed similar effects (17–20 eggs/female). The stronger effect of the oral treatment on oviposition is coherent with the observed accumulation of oocytes in the ovaries of insects treated this way (Table 6) and could be a result of a differential tissue partition of the drug via absorption through the gut. At higher doses, this difference is possibly minimized by the saturation of transport and absorption pathways to the ovaries.

Interestingly, the three delivery strategies had similar effects on the hatching of eggs that were deposited by treated females. This suggests that the amount of TFM, which is absorbed by oocytes during its development in the ovaries, does not depend on the TFM pathway from the insect's surface to the hemolymph. An exception for this rule is the strong reduction in hatching at the higher dose in the impregnated surface treatment (3.6%), which could be explained by a cumulative effect of the drug. Nevertheless, TFM topical treatment resulted in smaller oocytes, when compared to controls or the oral treatment (Table 6). As the oral treatment resulted in an inhibition of oviposition and accumulation of oocytes in the ovaries, these effects could compensate each other since the drug would be absorbed by a higher number of oocytes. It has also been shown that, in* R. prolixus*, hemolymphatic injection of lufenuron, a chitin synthesis inhibitor, decreases the number of eggs laid, modifies eggs shape and color, and reduces the viability of eggs by the disruption of chitin synthesis during oogenesis [31]. In this respect, apparently different BPU substances have similar effects on oogenesis and egg development.

The delivery mode strongly influences the effects of TFM on egg hatching of treated eggs, with strong inhibition after the topical treatment and no effect during the impregnated surface treatment. This could be related to the permeability of the eggshell to TFM, which could change during embryo development. However, it seems more likely that the continuous contact treatment failed due to a smaller egg surface in direct contact with the drug. Additionally, in the topical treatment, TFM was applied to the top surface of the egg, to avoid diffusion of the drug into the paper, and in the continuous contact treatment the bottom surface of the egg was in contact with the paper. In this last condition, absorption of drug is possibly negligible, considering that capillarity and diffusion effects would not occur across the eggshell, which is impermeable. One interesting hypothesis is that in the impregnated surface treatment the drug might be susceptible to environmental breakdown along the experiment, but the observation that first-instar nymphs that were born from the exposed eggs die after few days (data not shown) clearly suggests that the TFM is not significantly degraded. This is coherent with previous studies showing a strong effect of TFM on* R. prolixus* nymph survival [32]. This persistence in the environment, suggested by the effects of TFM in the continuous contact treatment, is an interesting property considering future applications of the drug in control of triatomines in the field. Also, from all the delivery modes tested, the continuous contact treatment was the only one that better reproduced the application of insecticides in control programs.

After characterization of the impact of TFM on the reproductive fitness of* R. prolixus* adult females, we studied some biological and biochemical parameters to have a better understanding of the impact of this drug on the insect's physiology. Initially, we observed that TFM treatment resulted in lower amounts of chitin in adult carcasses. Unfortunately, we were not able to measure chitin content in internal organs as gut or fat body, due to low sensitivity of the colorimetric quantitation technique. In fact, we expected a minimal amount of chitin in internal organs, related to the presence of trachea only. In the gut, the major source of chitin is possibly the cuticle of the foregut (which is vestigial in triatomines) and hindgut, as hemipterans do not have a peritrophic membrane in the midgut.* R. prolixus* midgut shows only the presence of N-acetylglucosamine [67], and only recently the possible presence of chitin in this tissue has been proposed [68].

In general, treatment with TFM resulted in a decrease in the chitin content of the carcass, which suggests that even in adult stages the amount of chitin depends on a homeostatic balance between synthesis and degradation, which could reflect age-related developmental changes in the cuticle or trachea. However, it is interesting to notice that the effect of TFM on the chitin content of carcass depends on the delivery mode and on the reproductive status of females. For nonmated females, we observed a bigger reduction of carcass chitin contents with the topical treatment, while in mated females the oral treatment resulted in a stronger effect. This could be a result of the activation of metabolism after mating, with higher absorptive and transport rates between the gut, fat body, and ovaries during oogenesis.

As previously discussed, the oral TFM treatment had apparently no significant effect on insect diuresis and a lower toxicity, possibly due to better elimination through gut microbiota, feces, or Malpighian tubules. Nevertheless, topical treatment with TFM resulted in a slower weight loss after blood feeding, especially during the first 48 hours. This may be interpreted as a symptom of impaired diuresis, which was confirmed by inspection of internal anatomy, revealing a great distension of the hindgut in treated insects. The swollen hindgut of treated insects could be interpreted as a result of a detoxification response with higher activity of Malpighian tubules, leading to accumulation of water in this structure. However, it is not clear why the insect did not excrete the urine produced in the first days after blood feeding. This could be a negative feedback response related to optimization of water balance in an insect which detected a continuous xenobiotic challenge.

Interestingly, feeding insects with blood containing TFM resulted in a significant increase of spontaneous PO levels in the hemolymph 7 days after treatment. Considering that in those individuals the amount of chitin in the carcass is reduced, it is possible that these insects are more prone to infection due to a weakened epithelial barrier against pathogens, resulting in a more activated PPO system. Activation of PPO during bacterial or fungal infection is a conserved and well-documented phenomenon in insects [69], being also already described in* R. prolixus* [36, 70–72]. More studies are necessary to determine if the TFM effect on* R. prolixus* PPO system is a direct effect of the drug on the PPO cascade or if it is an indirect effect involving a more frequent invasion of microorganisms into the hemolymph. It is important to note that the absence of effect on total PO activity registered over time and after TFM treatment is coherent with the constitutive nature of the PPO gene in* R. prolixus* [36] and suggests that expression of this gene is not being affected. Regulation of posttranslational activation of the PPO protein might be responsible for the observed PO activity changes.

In summary, very low concentrations of TFM reduced longevity, oviposition, and hatching of eggs laid by treated* R. prolixus* adult females. In addition, the viability of eggs oviposited by untreated females was affected by TFM topical treatment. Administration of this drug induced major changes in oogenesis, carcass chitin content, diuresis, and PPO activation system, severely affecting the insect physiology and leading to a premature death of treated females.

Chemical insecticides currently constitute a major tool in control programs of vector populations. However, the intense use of several compounds has generated resistance on the target insects [6]. The search of improved technologies involves the development of environmentally safe substances with selective action against target vectors and low risk for nontarget organisms [73]. Growth regulators, especially BPU substances, represent a promising new ecofriendly strategy [3, 7, 27, 53, 64, 74–76]. Our data suggest that TFM and perhaps other chitin synthesis inhibitors should be considered as potential tools for integrated vector control programs against hematophagous triatomine species.

## Figures and Tables

**Table 1:** Evaluation of the oral treatment with TFM on *Rhodnius prolixus* adult female mortality and reproduction. Control insects were fully engorged on defibrinated rabbit blood only, whereas experimental groups were fed with defibrinated rabbit blood added with Starycide at concentrations from 0.1 to 10 *µ*L/mL. Superscript letters show differences between treated and control insects: ^a^ *p* < 0.05; ^b^ *p* < 0.01; ^c^ *p* < 0.001 (unpaired *t*-test). Values are means ± SD of at least three experiments with 10 insects each.

Groups	Mortality		Oviposition, number of eggs per female	Egg hatching (%)
	1 day after treatment (%)	30 days after treatment (%)		
Control	9 ± 3	19 ± 3	56 ± 1	70 ± 20
0.1 *µ*L/mL	23 ± 8^a^	50 ± 10^b^	30 ± 10^a^	66 ± 5
0.5 *µ*L/mL	30 ± 10^a^	63 ± 8^b^	33 ± 9^b^	≤16^c^
1 *µ*L/mL	43 ± 5^b^	70 ± 10^b^	40 ± 30^a^	9 ± 3^c^
10 *µ*L/mL	40 ± 10^b^	76 ± 8^b^	20 ± 10^c^	≤16^c^

**Table 2:** Evaluation of topical treatment with TFM on *Rhodnius prolixus* adult female mortality and reproduction. Doses from 0.1 to 10 *µ*L of Starycide per insect were applied directly to the dorsal surface of the abdomen immediately after feeding of experimental insects. Control insects received no TFM. Superscript letters show differences between treated and control insects: ^a^ *p* < 0.05; ^b^ *p* < 0.01; ^c^ *p* < 0.001 (unpaired *t*-test). Values are means ± SD of at least three experiments with 10 insects each.

Groups	Mortality		Oviposition, number of eggs per female	Egg hatching, treated females (%)
	1 day after treatment (%)	30 days after feeding (%)		
Control	6 ± 3	23 ± 3	60 ± 6	70 ± 20
0.1 *µ*L	50 ± 10^b^	86 ± 6^c^	56 ± 7	60 ± 20
0.5 *µ*L	73 ± 3^b^	93 ± 1^c^	40 ± 5^a^	26 ± 9^c^
1 *µ*L	76 ± 5^b^	96 ± 1^c^	23 ± 4^b^	13 ± 3^c^
10 *µ*L	90 ± 3^c^	96 ± 3^c^	20 ± 3^c^	9 ± 2^c^

**Table 3:** Evaluation of topical treatment with TFM on *Rhodnius prolixus* egg hatching. Doses from 0.1 to 1 *µ*L of Starycide per egg were applied directly to the egg surface. Control insects received no TFM. Superscript letters show differences between treated and control insects: ^a^ *p* < 0.05; ^b^ *p* < 0.01 (unpaired *t*-test). Values are means ± SD of at least three experiments with 10 eggs each.

Groups	Egg hatching (%)
Control	93 ± 3
0.1 *µ*L	60 ± 6^a^
0.5 *µ*L	50 ± 6^a^
1 *µ*L	10 ± 6^b^
10 *µ*L	ND

**Table 4:** Evaluation of the effect of *Rhodnius prolixus* continuous contact with TFM impregnated surface on adult female mortality and reproduction. Petri dishes were prepared by applying Starycide to obtain final concentrations of 0.1–10 *μ*L/cm^2^ at the bottom of each plate. Controls received no Starycide. Insects were then fully engorged on defibrinated rabbit blood and placed inside the Petri dishes. Superscript letters show differences between treated and control insects: ^a^ *p* < 0.05; ^b^ *p* < 0.01; ^c^ *p* < 0.001 (unpaired *t*-test). Values are means ± SD of at least three experiments with 10 insects each.

Groups	Mortality in		Oviposition, number of eggs per female	Egg hatching, treated females (%)
	1 day after treatment (%)	30 days after treatment (%)		
Control	6 ± 3	16 ± 1	59 ± 2	70 ± 20
0.1 *μ*L/cm^2^	36 ± 8^a^	86 ± 8^c^	43 ± 5^a^	60 ± 10
0.5 *μ*L/cm^2^	40 ± 8^a^	90 ± 6^c^	36 ± 5^b^	18 ± 3^c^
1 *μ*L/cm^2^	40 ± 10^a^	93 ± 8^c^	20 ± 4^c^	9 ± 3^c^
10 *μ*L/cm^2^	63 ± 6^b^	96 ± 3^c^	17 ± 1^c^	3.6 ± 0.3^c^

**Table 5:** Evaluation of the continuous contact treatment with TFM on *Rhodnius prolixus *egg hatching. Petri dishes were prepared by applying Starycide to obtain final concentrations of 0.1–10 *µ*L/cm^2^ at the bottom of each plate. Controls received no Starycide. Freshly deposited eggs were then placed inside the Petri dishes. Values are means ± SD of at least three experiments with 10 eggs each.

Groups	Egg hatching (%)
Control	87 ± 7
0.1 *µ*L/cm^2^	ND
0.5 *µ*L/cm^2^	100
1 *µ*L/cm^2^	93 ± 3
10 *µ*L/cm^2^	87 ± 9

**Table 6:** Effect of oral or topical TFM administration on oocyte development inside treated *Rhodnius prolixus* adult females. Control insects were fully engorged on defibrinated rabbit blood only, whereas experimental insects were fed with blood containing 3 *μ*L TFM/mL blood (oral) or directly received 3 *μ*L TFM on the dorsal surface of the abdomen immediately after feeding (topical). Insects were kept for 5 days and dissected and then oocytes were counted and measured with the aid of a stereomicroscope. Values are means ± SEM of at least three experiments with 10 insects each. Asterisk denotes differences between control and experimental groups (unpaired *t*-test, *p* < 0.05).

TFM treatment	Oocytes	
	Number	Length
Control	5 ± 1	1.95 ± 0.02
Oral	9 ± 2^*∗*^	1.87 ± 0.03
Topical	4 ± 2	1.78 ± 0.06^*∗*^

**Table 7:** Effect of oral or topical TFM treatment on carcass chitin contents of *Rhodnius prolixus* adult females. Control insects were fully engorged on defibrinated rabbit blood only, whereas experimental insects were fed with blood containing 3 *μ*L TFM/mL blood (oral) or directly received 3 *μ*L TFM on the dorsal surface of the abdomen immediately after feeding (topical). Insects were kept for 5 days and dissected and then carcasses were submitted to chitin quantitation. Values are means ± SEM of at least three experiments with 3 insects each. Asterisk marks denote differences between control and experimental groups (unpaired *t-*test, ^*∗*^ *p* < 0.05; ^*∗∗*^ *p* < 0.01); hashtag denotes differences between mated and nonmated groups (^#^ *p* < 0.01).

TFM treatment	Mated (*μ*g/insect)	Nonmated (*μ*g/insect)
Control	7105 ± 5^#^	6760 ± 60
Oral	4700 ± 400^*∗∗*^	5100 ± 400^*∗*^
Topical	6700 ± 500	4300 ± 500^*∗∗*^
